# A numerical study to determine the effect of ligament stiffness on kinematics of the lumbar spine during flexion

**DOI:** 10.1186/s12891-016-0942-x

**Published:** 2016-02-22

**Authors:** Michael Putzer, Stefan Auer, William Malpica, Franz Suess, Sebastian Dendorfer

**Affiliations:** Laboratory of Biomechanics, Ostbayerische Technische Hochschule Regensburg, Josef-Engert-Straße 9, Regensburg, 93053 Germany; Regensburg Center of Biomedical Engineering, OTH and University Regensburg, Josef-Engert-Straße 9, Regensburg, 93053 Germany; Ortho Kinematics, 110 Wild Basin Rd., Suite 250, Austin, 78746 TX USA

**Keywords:** Lumbar spine, Ligament stiffness, Musculoskeletal modeling, Biomechanics

## Abstract

**Background:**

There is a wide range of mechanical properties of spinal ligaments documented in literature. Due to the fact that ligaments contribute in stabilizing the spine by limiting excessive intersegmental motion, those properties are of particular interest for the implementation in musculoskeletal models. The aim of this study was to investigate the effect of varying ligament stiffness on the kinematic behaviour of the lumbar spine.

**Methods:**

A musculoskeletal model with a detailed lumbar spine was modified according to fluoroscopic recordings and corresponding data files of three different subjects. For flexion, inverse dynamics analysis with a variation of the ligament stiffness matrix were conducted. The influence of several degrees of ligament stiffness on the lumbar spine model were investigated by tracking ligament forces, disc forces and resulting moments generated by the ligaments. Additionally, the kinematics of the motion segments were evaluated.

**Results:**

An increase of ligament stiffness resulted in an increase of ligament and disc forces, whereas the relative change of disc force increased at a higher rate at the L4/L5 level (19 %) than at the L3/L4 (10 %) level in a fully flexed posture. The same behaviour applied to measured moments with 67 % and 45 %. As a consequence, the motion deflected to the lower levels of the lumbar spine and the lower discs had to resist an increase in loading.

**Conclusions:**

Higher values of ligament stiffness over all lumbar levels could lead to a shift of the loading and the motion between segments to the lower lumbar levels. This could lead to an increased risk for the lower lumbar parts.

## Background

Back muscles stabilize the spine and they are supported by ligaments which limit excessive intersegmental motions [1]. Several studies described the activity of ligaments during different kinds of motions. Various flexion movements were investigated using the myoelectric activity and a noticeable role was assigned to the passive elements such as ligaments [2]. The anterior and posterior longitudinal ligaments lack a lax region, hence, they are loaded for all functional loads and therefore provide spinal stability [3]. However, spinal injuries can reduce that stability and change the load sharing between ligaments and muscles.

During the last decade, simulations have been used quite intensively to study biomechanics of the lumbar spine. A lumbar spine model was developed [4] and used as a starting point by different researchers investigating several cases. For example, Han and his colleagues enhanced and validated the model to predict muscle forces and to determine the effects of body height and weight on lumbar spine loading [5, 6]. Additionally, they studied the effect of different stiffness on joint and muscle forces and discovered a large influence of ligament stiffness on individual muscle forces [7].

In order to get a more realistic musculoskeletal model an increased level of detail is needed and in fact this is part of current developments in musculoskeletal research. Both, spinal muscles and ligaments are necessary to understand spinal loading conditions and to accurately predict forces as well as moments. However, the influence of input parameters such as ligament stiffness is not easy to determine. Especially in the case of musculoskeletal models which involve muscle recruitment the mechanical model gets complex.

To identify mechanical properties of spinal ligaments, several in-vitro studies have been carried out [8–11]. The reported values varied widely as it was to be expected because of the variability between individuals. The aim of this study was to investigate the effect of different ligament stiffness on the kinematics and kinetics of a musculoskeletal lumbar spine model during a flexion movement.

## Methods

In this study, three different musculoskeletal models each with a detailed lumbar spine were used for simulations with the AnyBody Modeling System (AMS, V. 5.3 AnyBody Technology, Aalborg, Denmark). All inverse dynamics computations were conducted with a polynomial muscle recruitment of the third order [12]. In contrast to traditional inverse dynamics approaches rotational displacement in the lumbar joints were computed using force dependent kinematics (FDK) capabilities [13].

### Specimen data

The data of the fluroscopic measurements was provided by Ortho Kinematics, Inc. (Austin, Texas). It can be obtained by contacting the company (GMalcolmson@orthokinematics.com). All the study participants provided written informed consent for the study. The fluoroscopic recordings were produced during a controlled standing flexion-extension movement. As the motion normalizer bent the trunk, about 180 radiographs were captured and image analysis software identified the vertebrae on each frame. The x- and y-coordinates of the vertices which belonged to the lumbar vertebral bodies L3 to S1 were collected in a data file. This data was used to determine each centre of rotation between the motion segments L3/L4, L4/L5 and L5/S1 [14]. Afterwards, the locations were compared to literature data [15]. Additionally, the datasets provided body height and weight.

### The base spine model

**Fig. 1:**
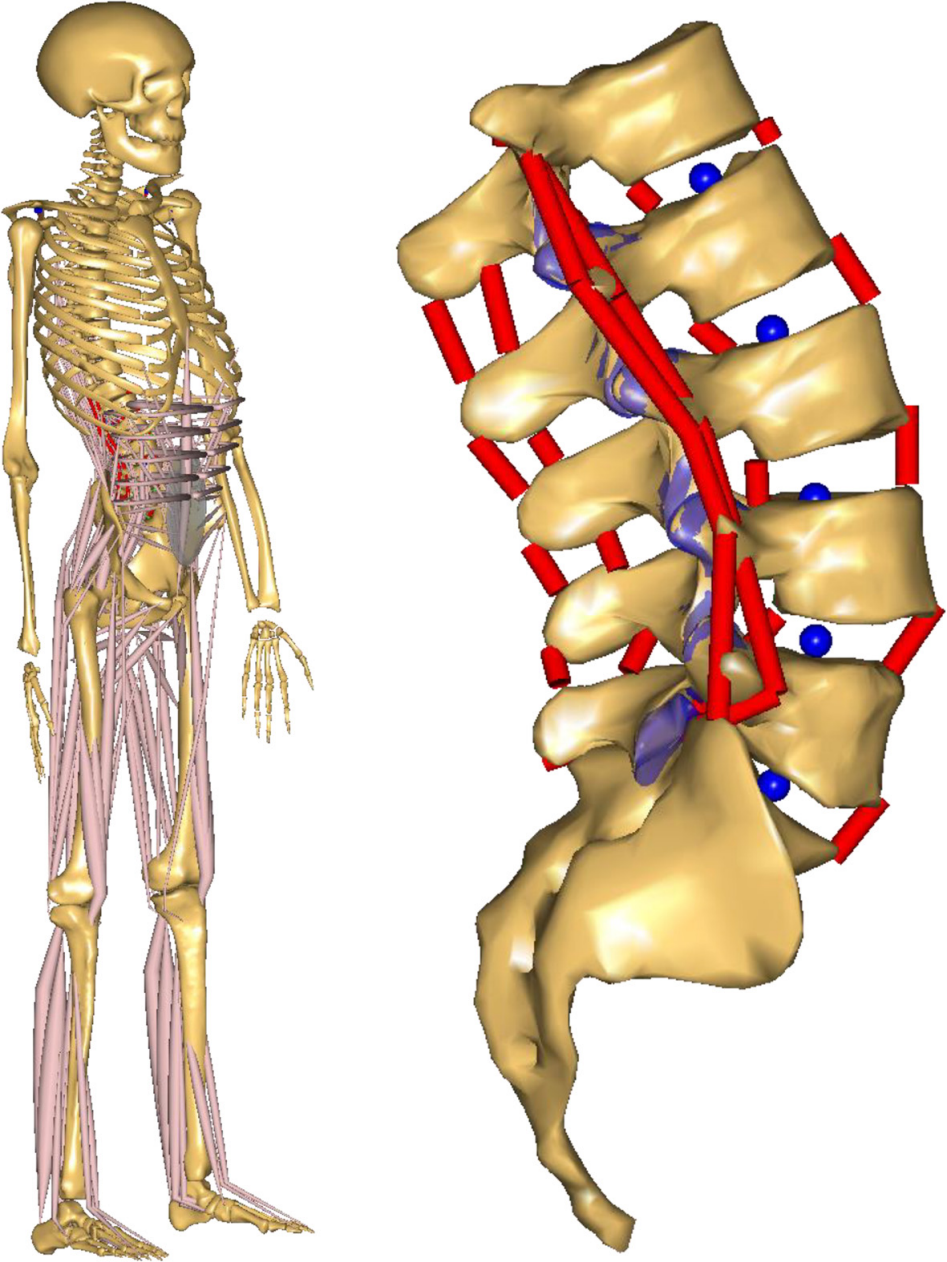
Musculoskeletal model. Full body musculoskeletal model (*left*) and an illustration of the lumbar spine with ligaments (*red*), facet surfaces (*violet*), and centers of rotation (*blue*) on the right side

The basis for the simulation model (Fig. 1) was available in the repository v. 1.4 (AMMR) accompanied by the AMS. It consisted of several rigid body components: skull, upper and lower extremities, pelvis and the spine. The spine included the cervical, thoracic and lumbar region, as well as the sacrum. The thoracic and cervical spine were modelled as a single lumped mass segment starting from T12 upwards while all vertebrae of the lumbar spine were modelled individually. Masses of the segments were distributed according to literature data [16].

The muscle apparatus was implemented as described by published data [4]. They were modelled as constant maximum force components and could only exert tensile forces.

The intervertebral discs were modelled as spherical joints with three rotational degrees of freedom and their stiffness properties were defined according to a linear relationship [17].

### Modifications to the base model

The lumbar ligaments (anterior and posterior longitudinal, interspinous, supraspinous, flavum, intertransverse) were defined as linear single force components, which generated a tensile force when stretched beyond their slack length. This length was defined in an upright standing posture. Stiffness and failure strain parameters were taken from literature data [8, 9]. Since the ligament implementation in the numerical model required the nominal strain, it was assumed to be 75 % of the failure strain.

The geometries of the lumbar vertebrae were morphed through segmentation of clinical computed tomographic images and each facet joint was designed with the help of a surface contact, where all contact forces between two articular processes were computed using contact area, stiffness and penetration depth. A first validation was performed with published data [18].

#### Generation of individual models

Three different models were generated for the purpose of this study. Therefore, body height and weight of each subject was implemented for a correct scaling of the body components. Additionally, the positions of the spherical joints between L3 to L5 were adapted according to the calculated centres of rotation from fluroscopy measurement. Moreover, the range of motion (ROM) of the simulation model was adjusted to the range of motion from the experiments.

### Variation of ligament stiffness

The values and ranges of failure strain and stiffness parameters for the ligaments were taken from an in-vitro experiment [9]. Each ligament stiffness was varied in eleven equal steps with a step size of 10 % using mean values and standard deviations as boundaries. Subsequently, eleven simulations each with an individual set of the stiffness matrix were executed for every single specimen. The data did not include properties of the intertransverse ligament. Therefore, mean values for this ligament were taken from another study [8]. Since these parameters were only determined in the T7/T8 and T9/T10 segment, no variation of the stiffness of the intertransverse ligament was conducted. The values and intervals for the stiffness matrix are shown in Table 1.

**Table 1:** Ligament stiffness matrix in N/mm with the according ranges of the intervals. The other sets were calculated by dividing the intervals into 11 equal steps

Ligament	T12-L1	L1-L2	L2-L3	L3-L4	L4-L5	L5-S1
ALL	32.9 ±20.9	32.4 ±13.0	20.8 ±14.0	39.5 ±20.3	40.50 ±14.3	13.20 ±10.2
PLL	10.0 ±5.5	17.1 ±9.6	36.6 ±15.2	10.6 ±8.5	25.8 ±15.8	21.8 ±16.0
ISL	12.1 ±2.6	10.0 ±5.0	9.6 ±4.8	18.1 ±15.9	8.7 ±6.5	16.3 ±15.0
SSL	15.1 ±6.9	23.0 ±17.3	24.8 ±14.5	34.8 ±11.7	18.0 ±6.9	17.8 ±3.8
LF	24.2 ±3.6	23.0 ±7.8	25.1 ±10.9	34.5 ±6.2	27.2 ±12.2	20.2 ±8.4
IT	50.0	50.0	50.0	50.0	50.0	50.0

The abbreviations are as follows: *ALL* anterior longitudinal, *PLL* posterior longitudinal, *ISL* intraspinous, *SSL* supraspinous, *LF* flavum, *IT* intertransverse

### Outcome variables

Influences of different sets of the ligament stiffness matrix were analysed by tracking ligament forces, joint forces and resulting moments generated by the ligaments with respect to the corresponding centre of rotation. Subsequently, the kinematics of the lumbar segments were evaluated by comparing the angles between the upper endplates.

## Results

Locations of centres of rotation were calculated and ligament forces, joint forces and resulting moments generated by ligaments were tracked and visualised in relation to the flexion movement in 33 simulations. The simulations consisted of 11 individual sets of the ligament stiffness matrix for three different subjects. Additionally, range of motion of the motion segments were determined by evaluating the angles between the according upper endplates.

### Centres of location

The calculation of centres of rotation resulted in good agreement with the published data [15] for subject one and two. Subject three showed centres of rotation beyond the boundaries of the literature data. Moreover, the calculation at the L5/S1 level produced locations with large deviations in comparison to the literature data. Therefore, the results for this level were not used in the models.

### Ligament and disc forces

**Fig. 2:**
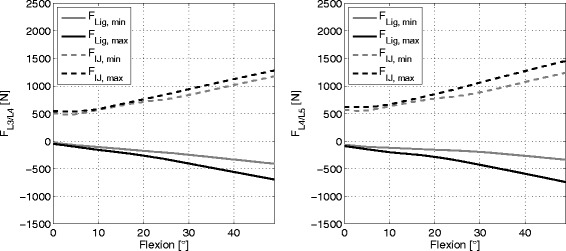
Ligament and intervertebral joint forces at minimum and maximums ligament stiffness. Ligament and intervertebral joint forces in superior-inferior direction at minimum and maximum ligament stiffness in the motion segments L3/L4 (*left*) and L4/L5 (*right*) for subject 1. The other subjects showed a similar behaviour

**Fig. 3:**
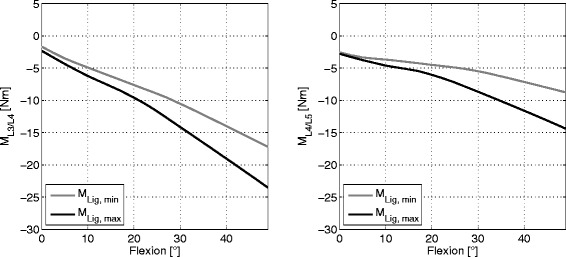
Changes of intervertebral joint forces for the different ligament stiffness. Intervertebral joint forces in the superior-inferior direction for the stiffness increase in a fully flexed posture

Figure 2 shows the ligament forces and the superior-inferior forces in the intervertebral disc for the L3/L4 and L4/L5 motion segment for minimal and maximal stiffness across the flexion movement. The joint reaction forces ranged from 498 N to 2112 N and the ligament forces ranged from 28 N up to 1026 N. The absolute joint forces were slightly larger in the L4/L5 segment as well as the relative force increase between minimum and maximum stiffness in a fully flexed posture (see Fig. 3). The corresponding mean increases with standard deviations (SD) are shown in Table 2.

**Table 2:** Relative increases of forces and moments

Lumbar level	Joint forces	Ligament forces	Moments
L3/L4	10 (4)	83 (12)	45 (8)
L4/L5	19 (3)	124 (6)	67 (2)

The table shows the mean values with its standard deviations in % of the relative increases of forces and moments between the minimum and maximum ligament stiffness during the fully flexed posture

### Moments generated by the ligaments

**Fig. 4:**
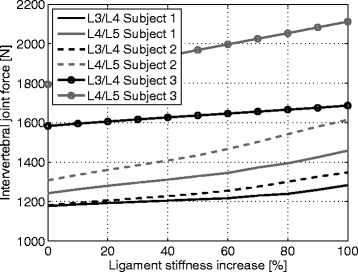
Resultant moments at minimum and maximum ligament stiffness. Resultant moments around the medio-lateral axis generated by the ligaments at minimum and maximum ligament stiffness in the motion segments L3/L4 (*left*) and L4/L5 (*right*) for subject 1. The other subjects showed a similar behaviour

The resulting moments generated by the ligaments with respect to the corresponding centre of rotation resulted in a total range of 2 to 26 Nm with higher absolute values in the L3/L4 segment compared to the L4/L5 segment. The highest values were obtained around the medio-lateral axis of the joint and are shown in Fig. 4. Similar to the behaviour of the forces the L4/L5 motion segment produced a larger relative increase of moments between minimum and maximum stiffness for all three subjects (see Table 2).

### Range of motion

**Fig. 5:**
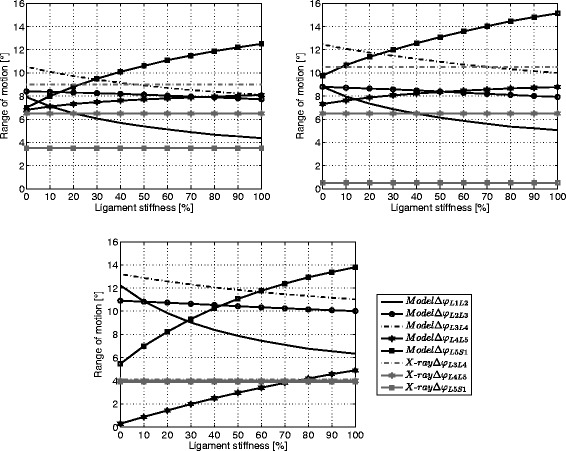
Range of motion of individual lumbar levels for the studied stiffness values. Range of motion calculated between the upper endplates of the motion segments as a function of the ligament stiffness for subject 1 (*top, left*), subject 2 (*top, right*) and subject 3 (*bottom*)

The ROM of each segment was calculated via the angles between the upper endplates of adjacent vertebrae. It is visualised as a function of the stiffness in Fig. 5 (0 *%*= minimum ligament stiffness, 100 % = maximum ligament stiffness) in conjunction with the ROM of the fluoroscopy data. The changes in L3/L4 of subjects one and two for the model (*ModelΔφ*_*L*3*L*4_) and the fluoroscopy data (*X*- *rayΔφ*_*L*3*L*4_) agree well at mean ligament stiffness. Furthermore, the ROM of the L4/L5 motion segment for the model (*ModelΔφ*_*L*4*L*5_) and the experiment (*X*- *rayΔφ*_*L*4*L*5_) match well regarding subject one and two, especially at minimum stiffness. However, a certain discrepancy was determined for the ROM at L5/S1 (*ModelΔφ*_*L*5*S*1_ and *X*- *rayΔφ*_*L*5*S*1_) for all three subjects.

Finally, the ROM of the upper motion segments (*ModelΔφ*_*L*1*L*2_, *ModelΔφ*_*L*2*L*3_, *ModelΔφ*_*L*3*L*4_) of all three subjects decreased with increasing ligament stiffness. In contrast, the lower segments (*ModelΔφ*_*L*4*L*5_, *ModelΔφ*_*L*5*S*1_) showed an increase of the range of motion with increasing stiffness.

## Discussion

The influence of different ligament stiffness on the motion behaviour of the lumbar spine was determined during a simulated flexion movement. Therefore, ligament forces, intervertebral joint forces as well as moments generated by the ligaments with respect to the corresponding centre of rotation were tracked. Subsequently, these simulations were evaluated with respect to the range of motion calculated from the angles between the upper endplates of the motion segments. Relative changes of forces and moments between minimum and maximum ligament stiffness during the fully flexed posture were determined.

While the simulations of subjects one and two showed comparable results, results for subject three revealed a different behaviour. For instance, the range of motion of the modelled L4/L5 motion segment (*ModelΔφ*_*L*4*L*5_) of subject three was almost zero at minimum ligament stiffness. This was probably caused by the large displacement of the centres of rotation for subject three in comparison to the experimental values [15] and the other subjects. Moreover, the simulated ROM of L5/S1 (*ModelΔφ*_*L*5*S*1_) did not agree very well with data of the fluoroscopic recordings (*X*- *rayΔφ*_*L*5*S*1_) for all subjects. This was probably caused by missing modifications of the corresponding centres of rotation however the calculations at this level did not return reasonable results. The small motion amplitude measured from the fluoroscopic recordings led to the defective calculations. This problem was also reported in literature [15].

However, the outcome of the simulations for all three subjects indicated that a rising ligament stiffness could cause a transfer of the motion and the loads to the lower lumbar segments. The growth rate of both result parameters was smaller in the upper lumbar parts. The deflection of the motion was clearly shown by the results for the ROM which decreased at the upper lumbar levels and at the same time increased at the lower levels. Even though, the absolute values for moments generated by ligaments were higher at the L3/L4 level compared to the L4/L5 level, the gain in intervertebral joint forces, ligament forces and moments induced from the ligament stiffness changes were higher at the lower segment.

These findings may be of particular interest for subject-specific simulations of the lumbar spine. They indicate that it is important to use a reasonable range of values for ligament stiffness to predict realistic results for the loading force and kinematics of the lumbar spine. Otherwise, simulations could lead to an overestimation of the kinematics and kinetics of the lower lumbar parts.

Additionally, the results could be relevant for degenerations connected with an increase of the stiffness for all lumbar levels. In these instances, this study implies that in case of a higher stiffness the lower lumbar motion segments may have to provide more motion and more resistance to increasing loads. As a result, discs within the lower lumbar range may potentially degenerate most rapidly.

There are some limitations to this work. The values of the ligament stiffness were altered uniformly over all lumbar levels neglecting changes in the distribution between the levels. Furthermore, the values were determined using only one publication [9] and to our knowledge, there are no studies on biomechanical properties of the intertransverse ligament at the lumbar level. Therefore, the data used in this study was utilised although the mechanical properties were determined in the T7/T8 and T9/T10 spinal level. Due to the lack of detailed information, mean values for all lumbar sections were transferred to the numerical model and nominal strain was assumed to be 75 % of the failure strain. Additionally, linear force-strain behaviour of the ligaments was supposed and no facet capsules were implemented in the model. Future work should include more subject specific data both, on the anatomical as well as the motion data to increase the significance of the data. Besides, the centres of location were not individualised at the spinal levels T12 to L3 due to lack of data from the fluoroscopic recordings.

## Conclusions

This study showed that higher values of ligament stiffness over all levels of the lumbar spine could lead to a shift of the loading to the lower lumbar levels. Furthermore, the kinematics of the lumbar spine showed a trend towards increased lower lumbar flexion. Overall, the results indicate that high stiffness values of the lumbar ligaments lead to an increased risk for the lower lumbar parts.

## Abbreviations

AMMR: anybody managed model repository
AMS: anybody modeling system
FDK: force dependent kinematics
ROM: range of motion
